# Corneal collagen cross-linking epithelium-on vs. epithelium-off: a systematic review and meta-analysis

**DOI:** 10.1186/s40662-021-00256-0

**Published:** 2021-09-01

**Authors:** Francesco D’Oria, Antonio Palazón, Jorge L. Alio

**Affiliations:** 1 Vissum Innovation, c/ Cabañal, 1, 03016 Alicante, Spain; 2 Division of Ophthalmology, Universidad Miguel Hernández, Alicante, Spain; 3 Section of Ophthalmology, Department of Basic Medical Science, Neuroscience and Sense Organs, University of Bari, Bari, Italy; 4 Department of Clinical Medicine, Miguel Hernández University, San Juan de Alicante, Spain

**Keywords:** Corneal collagen cross-linking, Keratoconus, Transepithelial CXL, Epithelium-off CXL, Epithelium-on CXL, Iontophoresis

## Abstract

**Background:**

The purpose of the study was to determine the advantages and disadvantages of epi-on corneal cross-linking (CXL) techniques compared with standard epi-off CXL.

**Methods:**

We searched MEDLINE and EMBASE for randomized controlled trials (RCTs) and non-randomized studies of interventions (NRSIs) and we evaluated the selected papers according to the Cochrane risk of bias tool. We considered, as primary outcomes, average Kmax flattening, changes in uncorrected and corrected distance visual acuity (UDVA and CDVA); as secondary outcomes, we considered changes in pachymetry values and endothelial cell density (ECD). We also investigated adverse events related to the treatments and treatment failure. Meta-analysis was conducted with a fixed or random-effects model using weighted mean difference (MD) with 95% confidence interval (CI) as the effect size.

**Results:**

A total of 15 studies were included and among these 15 trials, 9 were RCTs and 6 were NRSIs, but only 4 studies showed no high risk of bias and were included in this meta-analysis. Our analysis revealed significant postoperative differences in CDVA (MD = 0.07; 95% CI 0.04 to 0.10; *P* < 0.001), and no significative differences in UDVA, Kmax, central corneal thickness (CCT) and ECD (*P* > 0.05). Epi-on CXL protocol was found to be significantly less prompt to have risks of delay in epithelial healing (*P* = 0.035) and persistent stromal haze (*P* = 0.026).

**Conclusion:**

Epi-on CXL is as effective as epi-off CXL. Except for a higher significant improvement in CDVA with current epi-on protocols, our meta-analysis demonstrates that epi-on and epi-off CXL have comparable effects on visual, topographic, pachymetric, and endothelial parameters. Epi-on CXL has clinical advantages in terms of comfort and avoidance of complications as it reduces the risk of developing delay in epithelial healing and persistent stromal haze.

**Supplementary Information:**

The online version contains supplementary material available at 10.1186/s40662-021-00256-0.

## Background

Keratoconus is an ectatic corneal disorder affecting up to 1:375 in some populations, characterized by a progressive deformation and thinning of the cornea [1]. Disease detection is essential for improving the management of keratoconus patients as it can advance from mild changes to a severe loss of visual acuity that might require surgical approaches [2]. Several classification systems have been proposed to grade keratoconus as older ones e.g., Amsler–Krumeich classification system [3], although widely accepted, do not consider other variables such as the anterior corneal higher-order aberration (HOA) [4, 5].

Corneal cross-linking (CXL) was introduced in the late 1990s as a therapeutic approach to strengthen the biomechanical and biochemical properties of the cornea and the first clinical results were published by Wallensak et al. in 2003 [6]. The original CXL procedure is known as the “Dresden protocol” and is an epithelium-off procedure. In the standard technique, after anesthetizing the eye, the central 8 mm of the corneal epithelium is removed to expose the collagen-rich stroma and riboflavin solution (0.1% riboflavin-5-phosphate and 20% dextran T-500) is applied to the surface of the cornea both 30 min before irradiation and at 5 min intervals during a 30 min exposure to 370 nm UVA with a fluence of 3 mW/cm^2^ and a total irradiation dose of 5.4 J/cm^2^ [7]. The downside of epithelial removal is that it causes significant pain and discomfort in the early postoperative period; epi-off CXL carries a small risk of viral reactivation, haze, melting, infectious ulceration and the development of permanent stromal scars [8].

Considering these situations, several variations of the standard CXL procedure have been proposed since its introduction. Transepithelial or epi-on CXL is one such variation. Leaving the corneal epithelium intact should reduce pain and complications associated with epithelial debridement, such as infectious keratitis and could also lead to a shorter interruption of contact lens wear [8]. Considering transepithelial procedures, it should be considered that riboflavin is a large hydrophilic molecule that cannot penetrate an intact epithelium; moreover, the epithelium blocks about 20% of the UV illumination administered [9]. To improve riboflavin penetration into the stroma via the intact epithelium, several approaches have been used and investigated to encourage riboflavin penetration to the stroma [9].

There is, however, a paucity of studies that have been constructed to answer the clinical question of relative benefit for these procedures, and thus the evidence available for evaluation is limited. The main objectives of this systematic review and meta-analysis were to determine, in an evidence-based manner and using the currently available literature, the advantages and disadvantages of epi-on CXL techniques compared with traditional epi-off CXL and to discuss their indications.

## Methods

The review followed methods recommended by Cochrane and the Centre for Reviews and Dissemination [10] and is reported according to PRISMA guidelines [11]. Furthermore, it has been registered in the PROSPERO database (CRD42020156072), where the review methods were established prior to the beginning of the review analysis.

### Method of literature search

Two reviewers (FD and JLA) independently performed a systematic literature search in the MEDLINE/PubMed database and the EMBASE database from January 2014 to July 2021. This range was chosen to ensure that only contemporary and comparable procedures for CXL were included. The following keywords were used: “Keratoconus”, “Cross-Linking Reagents”, “Iontophoresis”, “Riboflavin”, “Epithelium”, “Ultraviolet Rays”, “Epi-On”, “Epi-Off”, “Epithelium-On”, “Epithelium-Off”. These were searched in the title and the abstract as well as MESH/EMTREE terms. Search terms are shown in Additional file 2. Only articles published in English, Italian, or Spanish with an available abstract were included in the systematic literature search (languages in which the authors had good command). The abstracts of related titles were reviewed, and the full articles retrieved if their title or abstract appeared to meet the objectives of this review. Studies were included if they met the inclusion criteria. Reference lists of the included papers were screened too. Moreover, we manually analyzed the following trial registries: clinicaltrials.gov and Cochrane Controlled Register of Trials as well as “grey literature”.

### Study selection, inclusion and exclusion criteria

All publications were screened by two authors (FD and JLA) and any disagreement was discussed by the two authors and resolved by consensus. The reports were screened according to the following selection criteria: (a) studies that compared the role of epi-on CXL to epi-off CXL for progressive keratoconus patients; (b) clinical trial studies; (c) both randomized controlled trials (RCTs) and non-randomized studies of interventions (NRSI), to generate evidence that will guide medical decisions [12]. All the studies that we reviewed were classified based on their scientific level of evidence according to the General Guidelines for Methodologies on Research and Evaluation of Traditional Medicine of the World Health Organization [13]. Only articles with a level Ib or IIa scientific evidence were selected. All the articles that were found were carefully reviewed to select those that reported original clinical data pre- and postoperatively. Data from previously reported cases included in different articles were omitted to avoid duplication of data. Articles on corneal collagen cross-linking combined with other treatments, such as topography-guided photorefractive keratectomy or intrastromal corneal ring segments were excluded. Experimental animal studies and ex vivo investigational reports were excluded from the review analysis. We also excluded all studies with a follow-up period of less than 12 months.

### Quality assessment

Two reviewers (FD and JLA) separately evaluated the studies based on the methods recommended in the Risk of Bias in Non-randomized Studies of Intervention (ROBINS-I) [10, 14] for cohort studies based on seven domains (confounders, selection of participants into the study, classification of interventions, deviations from the intended intervention, missing data, measurement of outcomes, and selection of the reported results). The “Cochrane risk of bias tool” [12] was used to assess the methodological quality of RCTs, by examining the following domains: random sequence generation, allocation concealment, blinding of participants and personnel, blinding of outcome assessment, incomplete outcome data, selective reporting, and other biases. The review authors looked for sources of funding for the studies included in the review. Any disagreement was discussed by the reviewers and resolved.

### Data extraction and clinical outcome

Two reviewers achieved consensus on which data to extract from the included studies (FD and JLA, followed the previous methods to achieve a consensus) and included the name of the first author, the year of publication, the trial location, the study design, the number of eyes, the type of CXL protocols used, the follow-up durations and outcome measures. In this review we considered, as the main outcome, average Kmax flattening, considering that the primary objective of CXL is to stabilize the underlying disease process, changes in uncorrected distance visual acuity (UDVA) and corrected distance visual acuity (CDVA), changes in HOAs and comatic aberrations, and depth of the demarcation line as an indirect treatment indicator of effectiveness. The secondary outcome parameters investigated in this study were changes in pachymetry values, either central corneal thickness (CCT) or corneal thickness at the thinnest point and endothelial cell density (ECD). We also investigated adverse events related to the treatments and treatment failure, such as keratoconus progression, loss of ≥ 2 lines of CDVA, delay in epithelial healing, persistent stromal haze, sterile infiltrates and infections.

### Statistical analysis

If all the included clinical characteristics were similar between groups, we think that it would be reasonable to combine these studies through a meta-analysis. Otherwise, a descriptive analysis will be carried out. If we performed the meta-analysis with our extracted data, following the AMSTAR-2 (Assessment of Multiple Systematic Reviews) checklist [15], we will only combine those works with no high risk of bias. For continuous outcomes, the weighted mean difference (MD) will be calculated for obtaining the absolute changes. This calculation will be carried out through a random-effects models, unless there is significant heterogeneity or if we have less than three studies [16]. Heterogeneity across studies will be estimated by using Cochran's Q test and I^2^, considering its presence when I^2^ > 50% and/or *P*-value > 0.10.

If significant heterogeneity existed among trials, we would explore sources of heterogeneity, using subgroup analysis and meta-regression models, so long as we have enough studies [16]. We will perform sensitivity analysis by repeating the calculations excluding groups of studies (those with unclear risk of bias and which have used iontophoresis) each time and compare the results obtained. A group of studies was considered influential if it varied the overall coefficient by at least 10%. Finally, we will analyze the asymmetry of funnel plots with the Egger test when we obtain at least 10 studies [17].

We set type I error at 5% and for each relevant parameter, its associated confidence interval (CI) was calculated. The statistical software for this meta-analysis was R 3.3.3 through the meta for package (Meta-Analysis Package for R).

## Results

### Systematic literature search

**Fig. 1:**
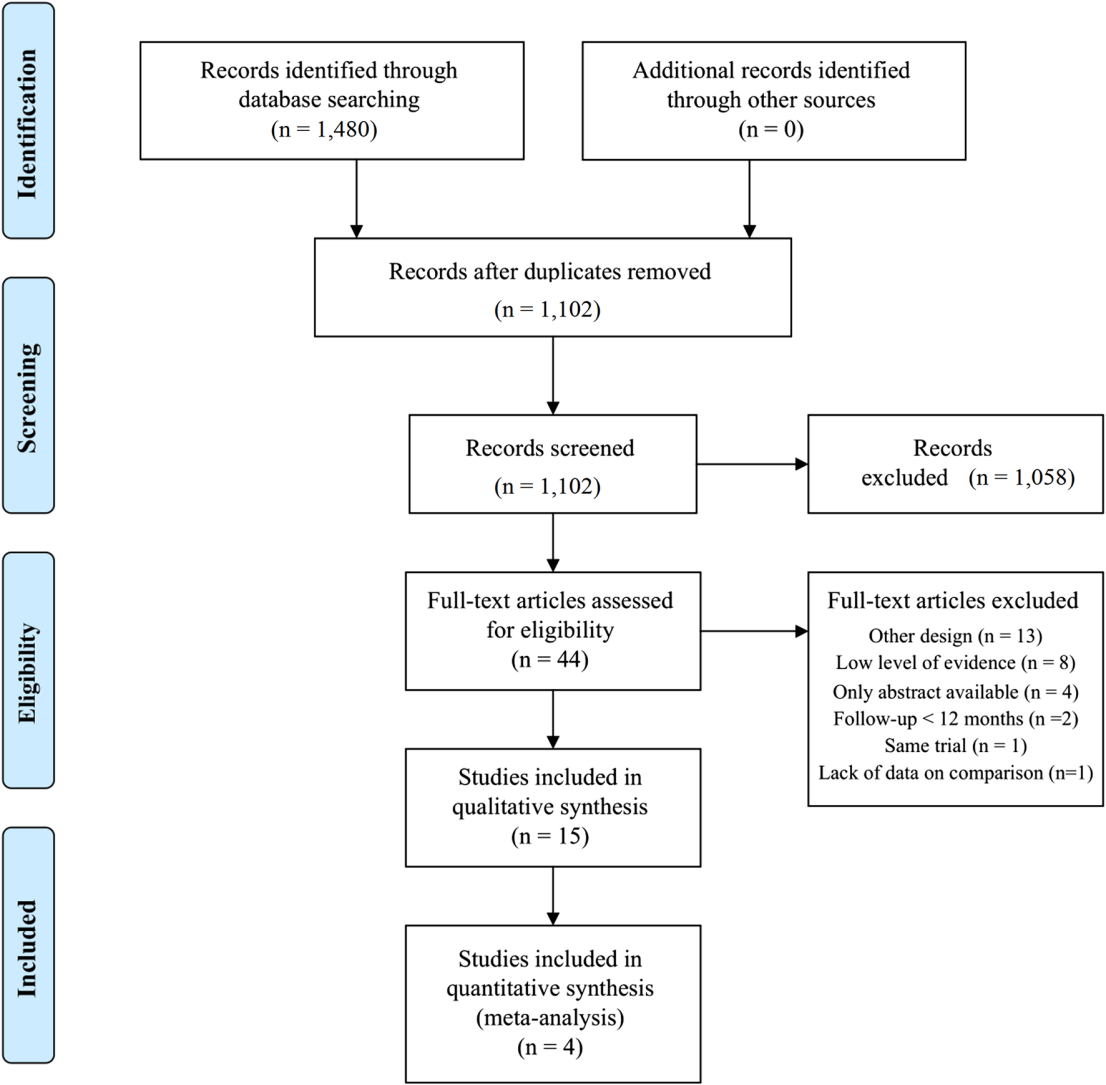
Flow chart of eligible papers used in the meta-analysis (PRISMA statement). PRISMA, preferred reporting items for systematic reviews and meta-analyses

This study identified 1102 publications after a systematic literature search and after the removal of duplicates as shown in the PRISMA flowchart (Fig. 1). Forty-four potentially relevant articles were identified and accessed in full text, 29 were excluded due to other design [18–30], level IIb or less of evidence [31–38], only abstract available [39–42], follow-up of less than 12 months [43, 44], same trial but with a shorter follow-up [45], and lack of data on comparison [46]. Fifteen relevant articles were included [47–61].

### Characteristics of included studies

We identified a total of 15 studies where epi-on CXL outcomes of progressive keratoconus patients were compared to epi-off CXL outcomes. Among these 15 trials, 9 were RCTs [48–50, 54, 55, 57, 59–61] and 6 were NRSIs [47, 51–53, 56, 58]. The country, study design, population and sample size, comparison, CXL regimen, outcome measures and follow-up of each clinical study were meticulously reviewed and summarized in Table 1. There were 511 eyes included in the standard epi-off CXL group and 574 eyes included in the epi-on CXL group. Studies were conducted in Italy, Jordan, Egypt, France, Peru, Netherlands, USA, Turkey, Russia and Saudi Arabia and were reported between 2015 and 2019. The duration of the follow-up ranged from 12 to 36 months. The study by Goodefroji et al. [54] derived data from a previously published RCT [61] to further investigate the development of HOAs and their effects on visual acuity, and thus only data concerning HOAs were extracted and included in the outcome measures (Tables 2, 3 and 4).

**Table 1:** Characteristics of the 15 studies included in the review

Authors, year	Country	Design	Population/Sample size	Comparison	Epi-on CXL protocol	Outcomes measures	Follow-up
Vinciguerra et al. 2019 [47]	Italy	Prospective comparative non-randomized clinical study	Adult with progressive keratoconuss /n = 60	20 S-CXL 20 I-CXL 20 I-SCXL	I-CXL: Constant current set at 1 mA for 5 min, Ricrolin + , UVA 10 mW/cm^2^ for 9 min	CDVA, refraction, topography, HOAs, pachymetry, ECD	24 months
Lombardo et al. 2019 [48]	Italy	Prospective unmasked randomized controlled trial	Adult with progressive keratoconus / n = 34	12 standard CXL 22 T-ionto CXL	T-ionto CXL: Current intensity set at 1.0 mA for 5 min, Ricrolin + , UVA 10 mW/cm^2^ for 9 min	UDVA, CDVA, refraction, contrast sensitivity, topography ECD, pachymetry, HOAs	24 months
Al Zubi et al. 2019 [49]	Jordan	Randomized control trial	Adult with progressive keratoconus / n = 80	40 epi-off CXL 40 transepithelial CXL	Transepithelial CXL: 0.1% riboflavin in 20% dextran for 30 min, UVA 3 mW/ cm^2^ for 30 min	UDVA, CDVA, pachymetry, topography	12 months
Iqbal et al. 2019 [50]	Egypt	Prospective multicenter controlled trial	Pediatric with progressive keratoconus / n = 271	91 S-CXL 92 A-CXL 88 T-CXL	T-CXL: 0.25% riboflavin, benzalkonium chloride and hydroxypropyl methylcellulose for 4.5 min, riboflavin 0.22% for 6 min, UVA 45 mW/cm^2^ for 5:20 min (pulsed)	UDVA, CDVA, refraction, topography, pachymetry	24 months
Rossi et al. 2018 [51]	Italy	Prospective clinical study	Adult with progressive keratoconus / n = 30	10 epi-off CXL 10 epi-on CXL 10 I-CXL	Epi-on CXL: Ricrolin TE for 30 min, UVA 3 mW/ cm^2^ for 30 min I-CXL: Current intensity set at 1.0 mA for 5 min, Ricrolin + , UVA 10 mW/cm^2^ for 10 min	UDVA, CDVA, refraction, topography, HOAs, pachymetry, ECD	12 months
Jouve et al. 2017 [52]	France	Prospective observational non-randomized clinical study	Adult with progressive keratoconus / n = 80	40 C-CXL 40 I-CXL	I-CXL: Current intensity set at 1.0 mA for 5 min, Ricrolin + , UVA 10 mW/cm^2^ for 9 min	CDVA, topography, pachymetry, confocal microscopy, demarcation line depth	24 months
Henriquez et al. 2017 [53]	Peru	Prospective cohort study	Pediatric with progressive keratoconus / n = 61	25 epi-off CXL 36 A-epi-on CXL	A-epi-on CXL: 0.25% riboflavin with 1.0% phosphate hydroxypropyl methylcellulose with 0.007% benzalkonium chloride for 30 min, UVA 18 mW/cm^2^ for 5 min	UCVA, BCVA, refraction, pachymetry, topography	12 months
Godefrooij et al. 2017 [54]	Netherlands	Noninferiority randomized clinical trial	Adult with progressive keratoconus / n = 61	26 epi-off CXL 35 transepithelial CXL	Transepithelial CXL: Ricrolin TE for 30 min, UVA 3 mW/ cm^2^ for 30 min	UDVA, CDVA, refraction, HOAs, topography, pachymetry, ECD, demarcation line depth	12 months
Rush et al. 2017 [55]	USA	Prospective randomized controlled clinical trial	Adult with progressive keratoconus / n = 144	64 epi-off CXL 80 transepithelial CXL	Transepithelial CXL: Riboflavin 0.25%, hydroxypropyl methylcellulose 1.2% and benzalkonium chloride 0.01% for 30 min, UVA 3 mW/ cm^2^ for 30 min	BCVA, topography, pachymetry	24 months
Eraslan et al. 2017 [56]	Turkey	Prospective comparative non-randomized interventional study	Pediatric with progressive keratoconus / n = 36	18 epi-off CXL 18 epi-on CXL	Epi-on CXL: 0.25% riboflavin, 1.2% hydroxypropyl methylcellulose and 0.01% benzalkonium chloride for 30 min, UVA 3 mW/cm^2^ for 30 min	UDVA, CDVA, topography, HOAs, pachymetry, demarcation line depth	24 months
Bikbova et al. 2016 [57]	Russia	Randomized controlled clinical study	Adult with progressive keratoconus / n = 149	73 standard CXL 76 transepithelial CXL	Transepithelial CXL: Current intensity gradually increased from 0.2 to 1.0 mA for 10 min, Riboflavin 0.1%, UVA 3 mW/cm^2^ for 30 min	UDVA, CDVA, keratometry, topography, pachymetry, demarcation line depth, confocal microscopy	24 months
Vinciguerra et al. 2016 [58]	Italy	Prospective comparative non-randomized interventional study	Adult with progressive keratoconus / n = 40	20 S-CXL 20 I-CXL	I-CXL: Constant current set at 1 mA for 5 min, Ricrolin, UVA 10 mW/cm^2^ for 9 min	CDVA, refraction, topography, HOAs, pachymetry, ECD	12 months
Al Fayez et al. 2015 [59]	Saudi Arabia	Prospective randomized parallel-group trial	Adult with progressive keratoconus / n = 70	36 epi-off CXL 34 transepithelial CXL	Transepithelial CXL: Tetracaine 1% with benzalkonium chloride 0.02% every 2 min for 30 min, UVA 3 mW/cm^2^ for 30 min	UDVA, CDVA, pachymetry, ECD, topography, IOP	36 months
Rossi et al. 2015 [60]	Italy	Noninferiority randomized clinical trial	Adult with progressive keratoconus/ n = 20	10 epi-off CXL 10 transepithelial CXL	Transepithelial CXL: Ricrolin TE for 30 min, UVA 3 mW/ cm^2^ for 30 min	UDVA, CDVA, refraction, topography, HOAs, CCT, ECD	12 months
Soeters et al. 2015 [61]	Netherlands	Noninferiority randomized clinical trial	Adult with progressive keratoconus / n = 61	26 epi-off CXL 35 transepithelial CXL	Transepithelial CXL: Ricrolin TE for 30 min, UVA 3 mW/ cm^2^ for 30 min	UDVA, CDVA, refraction, topography, pachymetry, ECD, demarcation line depth	12 months

*A-CXL* accelerated epithelium-off cross-linking; *A-epi-on CXL* accelerated transepithelial corneal collagen cross-linking; *BCVA* best-corrected visual acuity; *CDVA* corrected distance visual acuity; *CXL* corneal collagen cross-linking; *ECD* endothelial cell density; *HOA* higher-order aberration; *I-CXL* iontophoresis corneal collagen cross-linking; *I-SCXL* iontophoresis with epithelium removal corneal collagen cross-linking; *S-CXL* standard corneal collagen cross-linking; *T-CXL* transepithelial epithelium-off cross-linking; *T-ionto CXL* transepithelial iontophoresis corneal cross-linking; *UDVA* uncorrected distance visual acuity

In all the included trials, the control group was treated according to the standard epi-off CXL (Dresden protocol): central epithelial debridement, 0.1% riboflavin for 30 min, UVA 3 mW/cm^2^ for 30 min, total energy dose 5.4 J/cm^2^

**Table 2:** Mean differences in the main outcomes and adverse events in randomized controlled trials after corneal collagen cross-linking (epi-on *vs.* epi-off)

Study or subgroup	Epi-off CXL			Epi-on CXL		
	Mean ± SD	n (%)	n	Mean ± SD	n (%)	n
Kmax (D)						
Lombardo et al. 2019^a^ [48]	− 1.5 ± 4.9	–	12	− 1.0 ± 3.7	–	22
Iqbal et al. 2019^b^ [50]	− 1.2 ± 1.0	–	91	0.9 ± 1.1	–	88
Rossi et al. 2015^b^ [60]	− 1.1 ± 2.1	–	10	− 1.1 ± 1.0	–	10
Soeters et al. 2015^b^ [61]	− 1.5 ± 2.0	–	26	0.3 ± 1.8	–	35
UDVA (logMAR)						
Lombardo et al. 2019 [48]	− 0.3 ± 0.3	–	12	− 0.3 ± 0.4	–	22
Iqbal et al. 2019 [50]	− 0.3 ± 0.1	–	91	0.2 ± 0.1	–	88
Rossi et al. 2015 [60]	− 0.1 ± 0.1	–	10	− 0.1 ± 0.1	–	10
Soeters et al. 2015 [61]	− 0.1 ± 0.4	–	26	− 0.1 ± 0.4	–	35
CDVA (logMAR)						
Lombardo et al. 2019 [48]	− 0.0 ± 0.1	–	12	− 0.1 ± 0.1	–	22
Al Zubi et al. 2019 [49]	− 0.1 ± 0.1	–	40	− 0.1 ± 0.1	–	40
Iqbal et al. 2019 [50]	− 0.2 ± 0.2	–	91	0.1 ± 0.1	–	88
Rush et al. 2017 [55]	− 0.2	–	64	− 0.1	–	80
Bikbova et al. 2016 [57]	− 0.0 ± 0.3	–	73	− 0.1 ± 0.5	–	76
Rossi et al. 2015 [60]	− 0.1 ± 0.0	–	10	− 0.2 ± 0.1	–	10
Soeters et al. 2015 [61]	− 0.1 ± 0.2	–	26	− 0.1 ± 0.21	–	35
Higher-order aberrations (µm)						
Lombardo et al. 2019 [48]	− 0.1 ± 0.9	–	12	1.0 ± 1.1	–	22
Godefrooij et al. 2017 [54]	− 0.2 ± 0.4	–	26	0.0 ± 0.5	–	35
Comatic aberrations (μm)						
Rossi et al. 2015 [60]	− 0.4 ± 1.2	–	10	− 0.7 ± 1.3	–	10
Demarcation Line (depth in µm at 1 month)						
Bikbova et al. 2016 [57]	287.0 ± 15.0	–	73	179.0 ± 18.0	–	76
Soeters et al. 2015 [61]	266.0 ± 64.0	–	26	0.0	–	35
Central corneal thickness (µm)						
Lombardo et al. 2019 [48]	5.0 ± 21.0	–	12	7.0 ± 27.0	–	22
Al Zubi et al. 2019 [49]	5.4 ± 12.5	–	40	6.7 ± 15.5	–	40
Bikbova et al. 2016 [57]	− 13.0 ± 37.2	–	73	− 6.7 ± 38.6	–	76
Rossi et al. 2015 [60]	1.8 ± 14.6	–	10	− 2.7 ± 37.3	–	10
Corneal thinnest point (µm)						
Iqbal et al. 2019 [50]	− 8.9 ± 14.9	–	91	− 6.7 ± 9.0	–	88
Soeters et al. 2015 [61]	− 4.0 ± 8.0	–	26	0.0 ± 12.0	–	35
ECD (cells/mm^2^)						
Lombardo et al. 2019 [48]	− 30.0 ± 368.0	–	12	− 33.0 ± 309.0	–	22
Bikbova et al. 2016 [57]	20.0 ± 91.0	–	73	17.0 ± 69.0	–	76
Rossi et al. 2015 [60]	− 31.4 ± 66.6	–	10	− 53.0 ± 202.4	–	10
Soeters et al. 2015 [61]	− 59.0 ± 284.2	–	26	11.0 ± 338.9	–	35
KC progression						
Lombardo et al. 2019 [48]	–	0 (0.0%)	12	–	2 (9.1%)	22
Iqbal et al. 2019 [50]	–	0 (0.0%)	91	–	25 (28.4%)	88
Bikbova et al. 2016 [57]	–	0 (0.0%)	73	–	1 (1.3%)	76
Al Fayez et al. 2015 [59]	–	0 (0.0%)	36	–	19 (55.9%)	34
Soeters et al. 2015 [61]	–	0 (0.0%)	26	–	8 (22.9%)	35
Delay in epithelial healing						
Iqbal et al. 2019 [50]	–	17 (18.7%)	91	–	0 (0.0%)	88
Rush et al. 2017 [55]	–	1 (1.6%)	64	–	0 (0.0%)	80
Bikbova et al. 2016 [57]	–	4 (5.5%)	73	–	0 (0.0%)	76
Soeters et al. 2015 [61]	–	2 (7.7%)	26	–	0 (0.0%)	35
Persistent stromal haze						
Lombardo et al. 2019 [48]	–	2 (16.7%)	12	–	0 (0.0%)	22
Al Zubi et al. 2019 [49]	–	4 (10.0%)	40	–	0 (0.0%)	40
Iqbal et al. 2019 [50]	–	2 (2.2%)	91	–	0(0.0%)	88
Bikbova et al. 2016 [57]	–	4 (5.5%)	73	–	0 (0.0%)	76
Soeters et al. 2015 [61]	–	1 (3.8%)	26	–	0 (0.0%)	35
Sterile infiltrates						
Soeters et al. 2015 [61]	–	1 (3.8%)	26	–	0 (0.0%)	35
Infection						
Rush et al. 2017 [55]	–	1 (1.6%)	64	–	0 (0.0%)	80
Soeters et al. 2015 [61]	–	1 (3.8%)	26	–	0 (0.0%)	35

*CXL* corneal collagen cross-linking; *CDVA* corrected distance visual acuity; *D* diopter; *ECD* endothelial cell density; *KC* keratoconus; Kmax maximum keratometry; *logMAR* logarithm of the minimum angle of resolution; *n (%)* absolute frequency (relative frequency); *SD* standard deviation; *UDVA* uncorrected distance visual acuity

^a^Combined Placido disk corneal topography and anterior segment optical coherence tomography (Visante Omni, Carl Zeiss Meditec AG)

^b^Scheimpflug imaging analysis (Oculus Pentacam GmbH, Wetzlar, Germany)

**Table 3:** Mean differences in the main outcomes and adverse events in non-randomized studies of interventions after corneal collagen cross-linking (epi-on *vs.* epi-off)

Study or subgroup	Epi-off CXL			Epi-on CXL		
	Mean ± SD	n (%)	n	Mean ± SD	n (%)	n
Kmax (D)						
Vinciguerra et al. 2019^a^ [47]	− 0.1 ± 4.6	–	20	− 0.4 ± 4.4	–	20
Jouve et al. 2017^b^ [52]	− 1.1 ± 4.2	–	40	0.2 ± 5.2	–	40
Henriquez et al. 2017^a^ [53]	− 0.9 ± 5.2	–	25	0.1 ± 5.3	–	36
Eraslan et al. 2017^a^ [55]	− 1.4 ± 2.6	–	18	− 0.6 ± 3.1	–	18
Vinciguerra et al. 2016^a^ [57]	− 1.0 ± 1.5	–	20	− 0.3 ± 1.9	–	20
UDVA (logMAR)						
Rossi et al. 2018 [51]	− 0.1 ± 0.2	–	10	− 0.2 ± 0.2*	–	10*
				− 0.1 ± 0.2^Þ^	–	10^Þ^
Henriquez et al. 2017 [53]	− 0.1 ± 0.4	–	25	− 0.1 ± 0.2	–	36
Eraslan et al. 2017 [55]	− 0.0 ± 0.1	–	18	− 0.1 ± 0.2	–	18
CDVA (logMAR)						
Vinciguerra et al. 2019 [47]	− 0.1 ± 0.1	–	20	− 0.1 ± 0.2	–	20
Rossi et al. 2018 [51]	− 0.1 ± 0.1	–	10	− 0.1 ± 0.0*	–	10*
				− 0.1 ± 0.1 ^Þ^	–	10^Þ^
Jouve et al. 2017 [52]	− 0.1 ± 0.1	–	40	− 0.1 ± 0.2	–	40
Henriquez et al. 2017 [53]	− 0.1 ± 0.1	–	25	− 0.1 ± 0.1	–	36
Eraslan et al. 2017 [55]	− 0.1 ± 0.1	–	18	− 0.1 ± 0.1	–	18
Vinciguerra et al. 2016 [57]	− 0.0 ± 0.1	–	20	− 0.1 ± 0.1	–	20
Higher Order Aberrations (µm)						
Vinciguerra et al. 2019 [47]	− 0.1 ± 0.3	–	20	− 0.7 ± 0.2	–	20
Vinciguerra et al. 2016 [57]	− 0.0 ± 0.2	–	20	− 0.3 ± 0.8	–	20
Comatic Aberrations						
Vinciguerra et al. 2019 [47]	− 0.3 ± 1.0	–	20	1.7 ± 0.3	–	20
Rossi et al. 2018 [51]	− 0.4 ± 1.2	–	10	− 0.7 ± 1.4*	–	10*
				− 0.7 ± 1.3 ^Þ^	–	10^Þ^
Eraslan et al. 2017 [55]	− 0.2 ± 0.3	–	18	− 0.1 ± 0.5	–	18
Vinciguerra et al. 2016 [57]	− 0.2 ± 0.3	–	20	− 1.2 ± 1.6	–	20
Demarcation Line (depth in µm at 1 month)						
Jouve et al. 2017 [52]	291.0 ± 61.0	–	40	216.0 ± 49.0	–	40
Eraslan et al. 2017 [55]	272.3 ± 28.6	–	18	136.6 ± 17.9	–	18
Central corneal thickness (µm)						
Rossi et al. 2018 [51]	− 2.9 ± 18.9	–	10	− 1.6 ± 35.5*	–	10*
				− 4.7 ± 30.2 ^Þ^	–	10^Þ^
Corneal Thinnest Point (µm)						
Vinciguerra et al. 2019 [47]	− 57.0 ± 103.0	–	20	5.0 ± 38.0	–	20
Henriquez et al. 2017 [53]	− 12.5 ± 40.2	–	25	1.5 ± 51.5	–	36
Eraslan et al. 2017 [55]	− 11.3 ± 14.9	–	18	− 8.8 ± 14.8	–	18
Vinciguerra et al. 2016 [57]	− 41.1 ± 35.3	–	20	1.0 ± 7.2	–	20
ECD (cells/mm^2^)						
Rossi et al. 2018 [51]	− 32.7 ± 99.3	–	10	− 46.1 ± 197.9*	–	10*
				− 27.0 ± 62.5^Þ^	–	10^Þ^
KC progression						
Jouve et al. 2017 [52]	–	3 (7.5%)	40	–	8 (20.0%)	40
Henriquez et al. 2017 [53]	–	3 (12.0%)	25	–	2 (5.6%)	36
Lost ≥ 2 lines CDVA						
Eraslan et al. 2017 [55]	–	0 (0.0%)	18	–	1 (5.6%)	18
Persistent stromal haze						
Henriquez et al. 2017 [53]	–	1 (0.1%)	25	–	0 (0.0%)	36
Eraslan et al. 2017 [55]	–	5 (27.8%)	18	–	0 (0.0%)	18
Sterile infiltrates						
Henriquez et al. 2017 [53]	–	1 (0.1%)	25	–	0 (0.0%)	36

*CXL* = corneal collagen cross-linking; *CDVA* = corrected distance visual acuity; *D* = diopter; *ECD* endothelial cell density; *KC* = keratoconus; *Kmax* = maximum keratometry; *logMAR* = logarithm of the minimum angle of resolution; *n (%)*= absolute frequency (relative frequency); SD = standard deviation; *UDVA* = uncorrected distance visual acuity

*Transepithelial CXL treatment study group

^Þ^Iontophoresis CXL treatment study group

^a^Scheimpflug imaging analysis (Oculus Pentacam GmbH, Wetzlar, Germany)

^b^Scanning slit technique (Orbscan IIz; Bausch & LombSurgical, Rochester, NY)

**Table 4:** Sensitivity analysis (mean differences) for the primary and secondary outcomes in randomized clinical trials assessing corneal collagen cross-linking (epi-on *vs*. epi-off)

Subgroup	MD	95% CI	*P*
Kmax flattening (D)			
Low risk of bias	− 1.69	− 2.62 – − 0.76	0.004*
With iontophoresis	− 0.50	− 3.67 – 2.67	0.757
UDVA (logMAR)			
Low risk of bias	− 0.05	− 0.20 – 0.10	0.491
With iontophoresis	− 0.01	− 0.23 – 0.21	0.930
CDVA (logMAR)			
Low risk of bias	0.06	− 0.004 – 0.118	0.069
With iontophoresis	0.05	− 0.01 – 0.11	0.127
CCT (μm)			
Low risk of bias	− 2.0	− 18.38 – 14.38	0.811
With iontophoresis	− 4.76	− 14.53 – 5.02	0.340
ECD (cells/mm^2^)			
Low risk of bias	− 48.82	− 180.79 – 83.15	0.468
With iontophoresis	3.0	− 22.86 – 28.86	0.820

*CCT* central corneal thickness; *CDVA* corrected distance visual acuity; *CI* confidence interval; *D* diopter; *ECD* endothelial cell density; *logMAR* logarithm of the minimum angle of resolution; *MD* mean differences; *UDVA* uncorrected distance visual acuity

All the results were obtained through fixed-effects models because we only had two studies to summarize. If the value of the summary effect is negative, epi-on corneal collagen cross-linking would have more mean differences. Otherwise, epi-off would have this condition

*indicates statistical significance

### Primary outcomes

#### Corneal keratometry

**Fig. 2:**
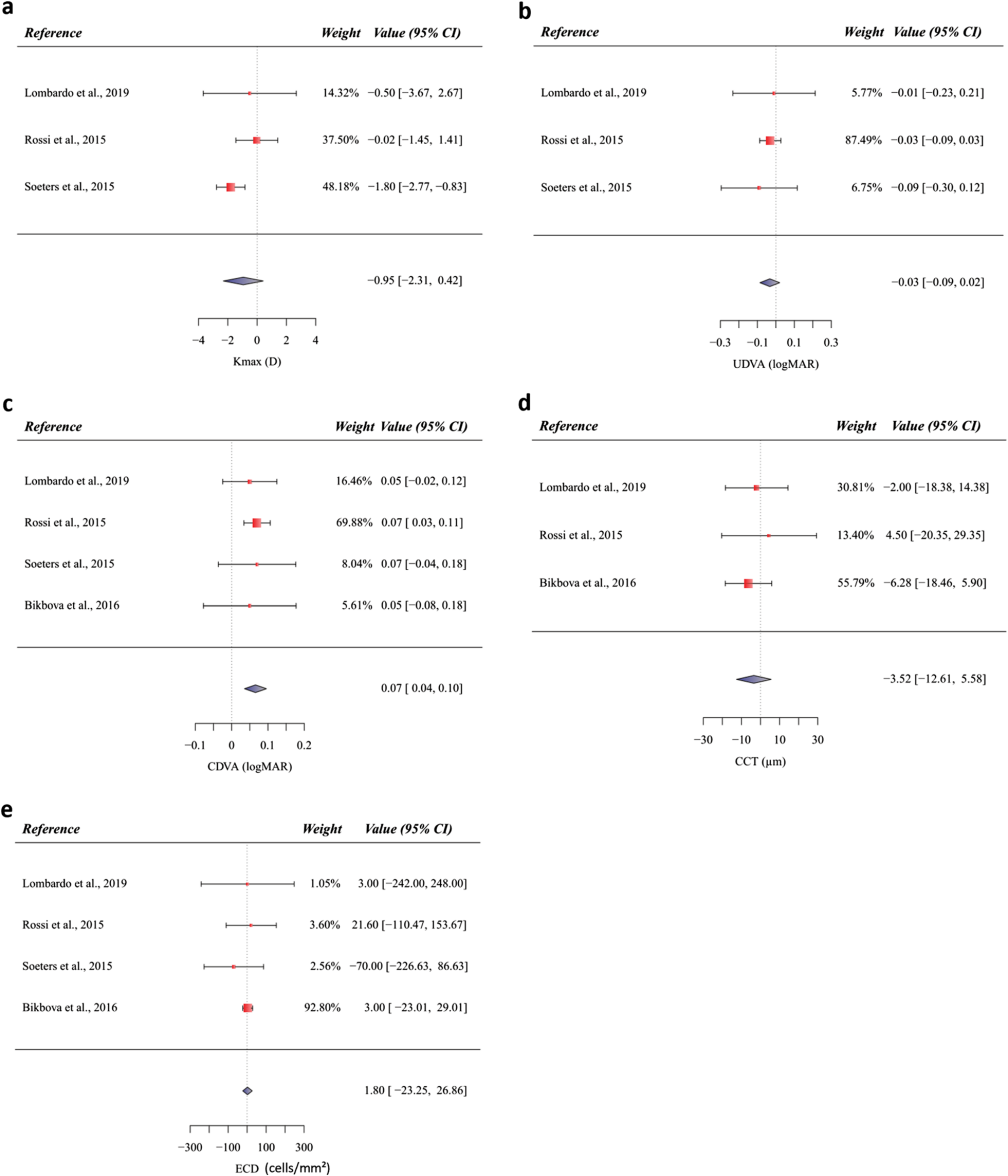
Forest plot of mean differences for the primary and secondary outcomes in randomized clinical trials assessing corneal collagen cross-linking (epi-on *vs*. epi-off): **a** standardized mean differences of change in maximum keratometry; **b** standardized mean differences of change in uncorrected distance visual acuity (UDVA); **c** standardized mean differences of change in corrected distance visual acuity (CDVA); **d** standardized mean differences of change in central corneal thickness (CCT); **e** standardized mean differences of change in endothelial cell density (ECD). The diamond at the bottom of each forest plot shows the result when all the individual studies are combined and averaged. If the outcome of interest favors the epi-on CXL, the diamond is moved to the right of the vertical line; if the outcome of interest favors the epi-off CXL, the diamond is moved to the left of the vertical line

Three studies compared the Kmax between the epi-on and epi-off CXL groups (Fig. 2) [48, 60, 61]. Among these studies (Fig. 2a), since there was evidence of heterogeneity (Q = 4.2615, df = 2, *P* = 0.1187; I^2^ = 54.28%), a random-effect model was used to calculate the pooled MD and its 95% CI (MD = − 0.95; 95% CI − 2.31, to 0.42; *P* = 0.174). The results of the sensitivity analysis (Table 4) showed a significant flattening of Kmax with epi-off CXL protocol considering only those with low risk of bias (MD = − 1.69; 95% CI − 2.62 to − 0.76; *P* = 0.004).

#### Visual acuity

Three studies compared the UDVA (logMAR) between the epi-on and epi-off CXL-treated groups [48, 60, 61]. As there was no obvious heterogeneity (Q = 0.3463, df = 2, *P* = 0.8410; I^2^ = 0%), a fixed-effect model was applied to calculate the pooled MD and its 95% CI (MD = − 0.03; 95% CI − 0.09 to 0.02; *P* = 0.228) (Fig. 2b). The exclusion of studies with unclear risk of bias or which have used iontophoresis from the full meta-analysis did not significantly alter the MD (Table 4), which ranged from − 0.05 (95% CI − 0.20 to 0.10; *P* = 0.491) to − 0.01 (95% CI − 0.23 to 0.21; *P* < 0.930).

Four studies compared the CDVA (logMAR) between the epi-on and epi-off CXL-treated groups [48, 57, 60, 61]. Since there was no obvious heterogeneity (Q = 0.2896, df = 3, *P* = 0.1187; I^2^ = 0%), the random-effect model was used to calculate the pooled MD and its 95% CI (MD = 0.07; 95% CI 0.04 to 0.10; *P* < 0.001) (Fig. 2c). Sensitivity analysis showed that there was not a main source of heterogeneity. The results remained unchanged after removing the studies with an unclear risk of bias (MD = 0.06; 95% CI − 0.004 to 0.118; *P* = 0.069) or with iontophoresis (MD = 0.05; 95% CI − 0.01 to 0.11; *P* = 0.127).

### Secondary outcomes

#### Pachymetry

Three studies compared the CCT between the epi-on and epi-off CXL groups (Fig. 2) [48, 57, 60]. There were no significant differences in CCT changes between the two groups (MD = 3.52; 95% CI − 12.61 to 5.58; *P* = 0.448) and no heterogeneity between studies (Q = 0.6306, df = 2, *P* = 0.7296; I^2^ = 0%) (Fig. 2d). The sensitivity analysis demonstrated that the result remained unchanged after the removal of the studies with an unclear risk of bias or performed with iontophoresis (MD = − 2.0 and − 4.76, respectively).

#### Endothelial Cell Density

Four studies compared the ECD between the epi-on and epi-off CXL-treated groups (Fig. 2) [48, 57, 60, 61]. There were no significant differences in ECD changes between the two groups (MD = 1.80; 95% CI − 23.25 to 26.86; *P* = 0.888) (Fig. 2e). Sensitivity analysis demonstrated that the result remained unchanged after removal of the study by Lombardo et al. [48] (MD = 3.0; 95% CI  − 22.86 to 28.86; *P* = 0.468), but the result did change after the removal of the studies with an unclear risk of bias (MD = − 48.82; 95% CI − 180.79 to 83.15; *P* = 0.820). Potential publication bias for both the primary and secondary outcomes was not assessed due to the limited number of studies available for review.

### Adverse events and treatment failure

Risk differences (RD) for adverse events and assessment of treatment failure in RCTs included in the quantitative analysis were assessed (Table 5). Epi-on CXL protocols were found to be significantly less prompt to have risks of delay in epithelial healing (RD = 0.049; 95% CI 0.003 to 0.0946; *P* = 0.035) and persistent stromal haze (RD = 0.0525; 95% CI 0.0063 to 0.0986; *P* = 0.026), and no significant for keratoconus progression (RD = − 0.025; 95% CI − 0.059 to 0.008; *P* = 0.141), sterile infiltrates (RD = 0.0036; 95% CI − 0.0206 to 0.0279; *P* = 0.768) and infection (RD = 0.0036; 95% CI − 0.0206 to 0.0279; *P* = 0.768). All results were obtained through fixed-effects models due to the lack of heterogeneity.

**Table 5:** Risk differences for the adverse effects and treatment failures in randomized clinical trials assessing corneal collagen cross-linking (epi-on *vs*. epi-off)

Adverse effect	RD	95% CI	*P*
Keratoconus progression	− 0.025	− 0.059 – 0.008	0.141
Delay in epithelial healing	0.049	0.0030 – 0.0946	0.035*
Persistent stromal haze	0.0525	0.0063 – 0.0986	0.026*
Sterile infiltrates	0.0036	− 0.0206 – 0.0279	0.768
Infection	0.0036	− 0.0206 – 0.0279	0.768

*CI* confidence interval; *RD * risk difference

If the value of the summary effect is negative, epi-on corneal collagen cross-linking would have more risk of adverse effects. Otherwise, epi-off would have this condition. All the results were obtained through fixed-effects models due to the lack of heterogeneity

*Indicates statistical significance

### Quality of the studies

The risk of bias assessment of RCTs and NRSIs is summarized in Additional file 1. Performance bias existed in all RCTs because personnel blinding to the intervention is impossible, but the review authors judge that the outcome is not likely to be influenced by lack of blinding. There was unclear evidence of random sequence generation in two studies [50, 57] and high risk of bias in two other studies [49, 55]. The allocation concealment was unclear in two studies [49, 50]. A high risk of bias due to incomplete data was present in one study [50]. Three studies had high risk of bias [54, 55, 59] and two had unclear risk of bias [49, 60] in selective reporting. Overall, only two studies [48, 61] were of high quality with a low risk of bias, whereas two [57, 60] were of unclear risk of bias and five [49, 50, 54, 55, 59] were of low quality with a serious risk of bias.

All the NRSIs were at serious risk of bias due to confounding factors [47, 51–53, 56, 58]; moreover, Eraslan et al. [56] had a serious risk of bias in the selection of participants, Rossi et al. [51] had a serious risk of bias due to missing data and Jouve et al. [52] had a medium risk of bias in measurements of outcomes.

## Discussion

CXL is now widely used to prevent the progression of keratoconus by strengthening the biomechanics of the human corneal stroma. Since its development, several clinical studies have reported the effectiveness of CXL using different transepithelial protocols and have compared the efficacy of both treatments. This meta-analysis of RCTs and NRSIs aimed at investigating the outcomes comparing different epi-on CXL protocols with standard epi-on CXL, whereas the difference in the epi-on CXL protocols used by the various authors has led to the lack of definitive evidence as to which technique is preferable.

Fifteen studies with 1085 eyes were included in the qualitative synthesis and a total of 4 studies with 264 eyes were included in the quantitative synthesis. Because the key treatment objective is to stabilize the underlying disease process, corneal topography (Kmax) was considered one of the primary outcome measures. Based on our meta-analysis, Kmax decreased slightly more after the epi-off CXL procedure but did not reach a statistically significant difference compared with epi-on CXL. Although CXL treatment is not intended to improve visual acuity, the induced changes in corneal topography may result in such a consequent improvement. Based on our systematic review and meta-analysis, the impact of CXL on visual acuity is remarkably different. CDVA showed a significant improvement with epi-on CXL (MD = 0.07; 95% CI 0.04 to 0.10). Based on the MD, patients treated with epi-on CXL had a larger 0.07 logMAR CDVA improvement as compared with control subjects; nevertheless UDVA was not different between both techniques. We assumed that patients treated with CXL protocols that preserved epithelium, probably based on the fewer numbers of postoperative corneal haze, had a higher improvement of CDVA [61]. In our study, we also found that ECD and CCT had no significant changes at long-term follow-up.

A previous systematic review and meta-analysis in 2018 [62] investigated the effectiveness of conventional CXL and transepithelial CXL based on RCTs and revealed no significant differences from the pooled results for the UDVA, CDVA, K-steepest, or ECD. Similarly, Wen et al. [63] compared standard epithelium-off CXL and transepithelial CXL for treating keratoconus and showed no differences in UDVA, CDVA and Kmax outcomes at 1 year. However, neither of the two systematic reviews investigated the RD for adverse events related to the procedures as well as the treatment failure rate, and we are unaware of any other similar analysis in previous systematic reviews. Based on our analysis, a significant delay in epithelial healing and persistent stromal haze were found in epi-off CXL if compared to epi-on CXL (Table 5).

The standard epi-off CXL procedure in all included studies followed the Dresden protocol (central corneal epithelium was removed, riboflavin drops were instilled for 30 min, and eyes were irradiated with UVA for 30 min at an irradiance of 3 mW/cm^2^).

We found that the methodology of the epi-on CXL protocol varied among the included studies. To increase epithelial permeability, Al Fayez et al. [59] used benzalkonium chloride and tetracaine; Soeters et al. [61] and Rossi et al. [51, 60] used Ricrolin TE solution (SOOFT Italia); Al Zubi et al. [49] used 0.1% riboflavin in 20% dextran for 30 min; Iqbal et al. [50], Henriquez et al. [53], Rush et al. [55] and Eraslan et al. [56] used benzalkonium chloride and hydroxypropyl methylcellulose. Bikbova et al. [57] performed riboflavin soaking using an iontophoresis device with 1.0 mA/cm^2^ current density for 10 min to induce absorption. Lombardo et al. preceded iontophoresis by removal of the precorneal mucin layer with the intent to increase epithelial permeability to riboflavin [48]. An iontophoresis device was also applied by Vinciguerra et al. [47, 58], Rossi et al. [51, 60] and Jouve et al. [52]. However, the total energy density was equal among studies. The disagreement between the epi-on CXL protocols used in the different RCTs has led to a lack of definitive evidence which can be overcome by the scientific methodology applied in the current meta-analysis.

Studies conducted by Vinciguerra et al. [47, 58], Lombardo et al. [48] and Jouve et al. [52] used 10 mW/cm^2^ irradiation for 9 min, while the study conducted by Al Zubi et al. [49], Rossi et al. [51, 60], Rush et al. [55], Eraslan et al. [56], Bikbova et al. [57], Al Fayez et al. [59] and Soeters et al. [61] used a 3 mW/cm^2^ irradiation device for 30 min. Henriquez et al. [53] used an accelerated protocol to shorten 5 min of irradiation (18 mW/cm^2^), while Iqbal et al. [50] used a pulsed protocol of irradiation (UVA 45 mW/cm^2^ for 5:20 min). Therefore, the total irradiation dose was approximately 5.4 J/cm^2^ in each included study. The RCTs included in this study used the same irradiation energy–making this parameter homogeneous among all of them–and were compared according to whether the corneal epithelium was preserved or not.

Three of the 15 studies included in the current review treated pediatric patients with progressive keratoconus. Nevertheless, all the 4 RCTs included in the quantitative meta-analysis treated adult patients, and thus the results were not influenced by the patients' age.

The natural course of keratoconus can be long-lasting, with years of apparent stable keratometry readings after a period of latent progression. Only RCTs trials with adequate follow-up of at least one year have been included in this systematic analysis. Nevertheless, all the analyzed studies followed patients for less than 5 years. Consequently, questions about the long-term stability and efficacy of epi-on CXL compared to epi-off CXL beyond that period must be answered and RCTs comparing the long-term outcomes between these techniques are warranted.

Our study has several strengths. To the best of our knowledge, this is the first meta-analysis investigating the adverse events and treatment failure of conventional and transepithelial CXL for patients with keratoconus. Second, we update our review if compared to the previous systematic reviews on the topic: by including 3 RCTs and 1 NRSI published in 2019, we report the most updated evidence. Third, our quantitative evaluation was based only on prospective well-designed RCTs studies with no high risk of bias, increasing the consistency of the results.

This systematic review also has several limitations that should be considered. First, like any meta-analysis, there is variability between the methodologies used for epi-on CXL among studies. However, our sensitivity analysis did not significantly alter the results when these trials using transepithelial or iontophoresis techniques were excluded. Second, we acknowledge the small number of cases per trial however, the total number of cases included in the meta-analysis gives these analyses high power. Moreover, among the 9 RCTs identified, three were published in open access journals that may or may not have limited the veracity of the data but these were not included in the meta-analysis thereby minimizing their influence on the results presented.

## Conclusion

In conclusion, our systematic review and meta-analysis of the literature documenting the comparative efficacy of epi-on *vs.* epi-off CXL in halting the progression of keratoconus found that both protocols are equally effective. According to the studies included in the quantitative meta-analysis, the rate of keratoconus progression is not significantly different with the two protocols. The published evidence indicates that, except for a larger significant improvement in CDVA with current epi-on CXL protocols, the analysis of well-designed comparative studies available demonstrates that epi-on and epi-off CXL have comparable effects on visual, topographic, pachymetric, and endothelial parameters at 1–2 years after surgery in adult patients. Epi-on CXL protocols, by preserving the epithelium, offers a significant reduction in epithelial healing delay and stromal haze formation and a faster postoperative recovery. Current evidence leaves the field open for final, confirmatory, well-designed unbiased RCTs to confirm the long-term efficacy and safety outcomes as well as similarities and differences between the epi-on and epi-off CXL techniques.

## Supplementary Information

**Additional file 1.** Quality evaluation and bias assessment of the included studies. **a** Quality evaluation and bias assessment of randomized controlled trials; **b** Quality evaluation and bias assessment of non-randomized studies of interventions.

**Additional file 2. **Search terms used in the MEDLINE/PubMed database and the EMBASE database.

## Data Availability

All data analyzed during this study are included in this published article and its Additional files.

## Abbreviations

CCT: Central corneal thickness
CI: Confidence interval
CXL: Corneal collagen cross-linking
CDVA: Corrected distance visual acuity
ECD: Endothelial cell density
HOA: Higher-order aberration
MD: Mean difference
NRSI: Non-randomized studies of interventions
RCT: Randomized controlled trials
RD: Risk difference
UDVA: Uncorrected distance visual acuity
