# Supplementary material for: Corneal collagen cross-linking epithelium-on vs. epithelium-off: a systematic review and meta-analysis
Source: Eye Vis (Lond). 2021 Sep 1;8:34. doi: 10.1186/s40662-021-00256-0 (PMC8465763; doi:10.1186/s40662-021-00256-0)
Supplement: Supplementary file 1 — Additional file 1. Quality evaluation and bias assessment of the included studies. a Quality evaluation and bias assessment of randomized controlled trials; b Quality evaluation and bias assessment of non-randomized studies of interventions. [file 40662_2021_256_MOESM1_ESM.pdf]

a

	Lombardo et al., 2019	Al Zubi et al., 2019	Iqbal et al., 2019	Godefrooij et al., 2017	Rush et al., 2017	Bikbova et al., 2016	Al Fayez et al., 2015	Rossi et al., 2015	Soeters et al., 2015
Random sequence generation	Low risk	High risk	Unclear risk	Low risk	High risk	Unclear risk	Low risk	Low risk	Low risk
Allocation concealment	Low risk	Unclear risk	Unclear risk	Low risk	Low risk	Low risk	Low risk	Low risk	Low risk
Blinding of participants and personnel	High risk	High risk	High risk	High risk	High risk	High risk	High risk	High risk	High risk
Blinding of outcome assessment	Low risk	Low risk	Low risk	Low risk	Low risk	Low risk	Low risk	Low risk	Low risk
Incomplete outcome data	Low risk	Low risk	High risk	Low risk	Low risk	Low risk	Low risk	Low risk	Low risk
Selective reporting	Low risk	Unclear risk	Low risk	High risk	High risk	Low risk	High risk	Unclear risk	Low risk
Other bias	Low risk	Low risk	Low risk	Low risk	Low risk	Low risk	Low risk	Low risk	Low risk

b

	Vinciguerra et al., 2019	Rossi et al., 2018	Jouve et al., 2017	Eraslan et al., 2017	Henriquez et al., 2017	Vinciguerra et al., 2016
Bias due to confounding	Serious risk	Serious risk	Serious risk	Serious risk	Serious risk	Serious risk
Bias in selection of participants into the study	Low risk	Low risk	Low risk	Serious risk	Low risk	Low risk
Bias in classification of intervention	Low risk	Low risk	Low risk	Low risk	Low risk	Low risk
Bias due to deviation from intended intervention	Low risk	Low risk	Low risk	Low risk	Low risk	Low risk
Bias due to missing data	Low risk	Serious risk	Low risk	Low risk	Low risk	Low risk
Bias in measurement of outcomes	Low risk	Low risk	Moderate risk	Low risk	Low risk	Low risk
Bias in selection of the reported results	Low risk	Low risk	Low risk	Low risk	Low risk	Low risk
Overall risk of bias	Serious risk	Serious risk	Serious risk	Serious risk	Serious risk	Serious risk
