# Supplementary material for: Corneal collagen cross-linking epithelium-on vs. epithelium-off: a systematic review and meta-analysis
Source: Eye Vis (Lond). 2021 Sep 1;8:34. doi: 10.1186/s40662-021-00256-0 (PMC8465763; doi:10.1186/s40662-021-00256-0)
Supplement: Supplementary file 2 — Additional file 2. Search terms used in the MEDLINE/PubMed database and the EMBASE database. [file 40662_2021_256_MOESM2_ESM.docx]

MEDLINE:

(“Keratoconus”[mesh])

AND

(“Cross-Linking Reagents”[mesh] OR “Iontophoresis”[mesh] OR “Riboflavin”[mesh] OR “Epithelium”[mesh] OR “Ultraviolet Rays”[mesh] OR “Epi-On”[title/abstract] OR “Epi-Off”[title/abstract] OR “Epithelium-On”[title/abstract] OR “Epithelium-Off”[title/abstract])

Filters activated: Abstract, Publication date from 2014/01/01, English, Italian, Spanish. Clear all to show 1435 items.

01-07-2021

EMBASE:

('keratoconus'/exp OR 'keratoconus') AND ('cross linking reagent'/exp OR 'cross linking reagent' OR 'iontophoresis'/exp OR 'iontophoresis' OR 'riboflavin'/exp OR 'riboflavin' OR 'epithelium'/exp OR 'epithelium' OR 'ultraviolet radiation'/exp OR 'ultraviolet radiation' OR 'epi-on':ti,ab OR 'epi-off':ti,ab OR 'epithelium-on':ti,ab OR 'epithelium-off':ti,ab) AND ([english]/lim OR [italian]/lim OR [spanish]/lim) AND [abstracts]/lim AND [2014-2021]/py
